# e-PTSD: an overview on how new technologies can improve prediction and assessment of Posttraumatic Stress Disorder (PTSD)

**DOI:** 10.1080/20008198.2018.1424448

**Published:** 2018-02-06

**Authors:** Alexis Bourla, Stephane Mouchabac, Wissam El Hage, Florian Ferreri

**Affiliations:** ^a^ Department of Psychiatry, Sorbonne Université, AP-HP, Hôpital Saint-Antoine, Service de Psychiatrie, Paris, France; ^b^ Clinique Psychiatrique Universitaire, CHRU de Tours, Université François-Rabelais de Tours, Tours, France

**Keywords:** Posttraumatic stress disorder, digital phenotype, Ecological Momentary Assessment, virtual reality, heart rate variability, new technologies, Artificial Intelligence, TEPT, fenotipo digital, Evaluación Ecológica Momentánea, realidad virtual, Variabilidad de la frecuencia cardiaca, Nuevas tecnologías, Inteligencia artificial, PTSD, 数码表型, 生态瞬时评估, 虚拟现实, 心率变异性, 新技术, 人工智能, • The impact of the new technologies on health professionals practice in PTSD care remains to be determined. • We conducted an overview on the technologies used for prediction and assessment of PTSD.• This review shows that it is possible to start building the PTSD digital phenotype.• These tools must therefore be explained and adapted to the different profiles of physicians and patients.

## Abstract

**Background**: New technologies may profoundly change our way of understanding psychiatric disorders including posttraumatic stress disorder (PTSD). Imaging and biomarkers, along with technological and medical informatics developments, might provide an answer regarding at-risk patient’s identification. Recent advances in the concept of ‘digital phenotype’, which refers to the capture of characteristics of a psychiatric disorder by computerized measurement tools, is one paradigmatic example.

**Objective**: The impact of the new technologies on health professionals practice in PTSD care remains to be determined. The recent evolutions could disrupt the clinical practices and practitioners in their beliefs, ethics and representations, going as far as questioning their professional culture. In the present paper, we conducted an extensive search to highlight the articles which reflect the potential of these new technologies.

**Method**: We conducted an overview by querying PubMed database with the terms [PTSD] [Posttraumatic stress disorder] AND [Computer] OR [Computerized] OR [Mobile] OR [Automatic] OR [Automated] OR [Machine learning] OR [Sensor] OR [Heart rate variability] OR [HRV] OR [actigraphy] OR [actimetry] OR [digital] OR [motion] OR [temperature] OR [virtual reality].

**Results**: We summarized the synthesized literature in two categories: prediction and assessment (including diagnostic, screening and monitoring). Two independent reviewers screened, extracted data and quality appraised the sources. Results were synthesized narratively.

**Conclusions**: This overview shows that many studies are underway allowing researchers to start building a PTSD digital phenotype using passive data obtained by biometric sensors. Active data obtained from Ecological Momentary Assessment (EMA) could allow clinicians to assess PTSD patients. The place of connected objects, Artificial Intelligence and remote monitoring of patients with psychiatric pathology remains to be defined. These tools must be explained and adapted to the different profiles of physicians and patients. The involvement of patients, caregivers and health professionals is essential to the design and evaluation of these new tools.

## Abstract (es)

**Contexto**: Las nuevas tecnologías podrían cambiar de manera profunda nuestra forma de entender los trastornos psiquiátricos incluyendo el trastorno de estrés postraumático (TEPT). Técnicas de imagen y biomarcadores, junto con desarrollos en informáticas médicas y tecnológicas, pueden proveer una respuesta en relación con la identificación de pacientes en riesgo. Avances recientes en el concepto de ‘genotipo digital’, que se refiere a la ‘captura’ de las características de un trastorno psiquiátrico mediante instrumentos de medición computarizada, es un ejemplo paradigmático de todo ello.

**Objetivo**: El impacto de las nuevas tecnologías en la practica de los profesionales de la salud en el ámbito del tratamiento del trastorno de estrés postraumático (TEPT), está aún por determinar. Las evoluciones recientes pueden trastornar o interrumpir tanto las prácticas clínicas como a los profesionales que las ejercen. También a sus conceptos éticos y a sus representaciones hasta el punto de poder poner en cuestión su cultura profesional. En este trabajo, hemos realizado una búsqueda intensa con el fin de poner de manifiesto los trabajos que mejor reflejan el potencial de estas nuevas tecnologías.

**Método**: Hemos realizado una búsqueda panorámica en la base de datos PubMed basándonos en títulos hasta Junio de 2017 que contenían los términos ‘[PTSD] [Posttraumatic stress disorder] AND [Computer] OR [Computerized] OR [Mobile] OR [Automatic] OR [Automated] OR [Machine learning] OR [Sensor] OR [Heart rate variability] OR [HRV] OR [actigraphy] OR [actimetry] OR [digital] OR [motion] OR [temperature] OR [virtual reality]’.

**Resultados**: hemos resumido la literatura sintetizada en dos categorías: predicción y evaluación (incluyendo dignóstico, discriminación y monitorización). Dos investigadores independientes realizaron el triaje, extrajeron los datos y determinaron la calidad de las fuentes. Los resultados se refieren y sintetizan narrativamente.

**Conclusiones**: este estudio muestra que muchos estudios están en marcha permitiendo empezar a construir un fenotipo digital de TEPT, usando datos pasivos obtenidos por sensores biométricos. Datos activos obtenidos a través de una Evaluación Momentánea Ecológica (EME), puede permitir evaluar a pacientes con TEPT para hacernos una idea de su severidad. El lugar que deben ocupar objetos conectados, inteligencia artificial y monitorización remota debe ser aún definido. Para poder ser utilizados, estos instrumentos deben ser por tanto explicados y adaptados a los diferentes perfiles de médicos y pacientes. La participación de los pacientes, cuidadores y de otros profesionales de la salud es esencial cara a diseñar y evaluar estos nuevos instrumentos.

## Abstract (zh)

背景: 新技术巨大地改变了我们对精神疾病的理解方式，包括对创伤后应激障碍（PTSD）。脑成像和生物标记，与技术和医学信息的发展一起，也许提供了一个识别有风险的病人的途径。最近关于‘数码表型’（指使用电脑化测量工具来‘捕捉’精神疾病特征）的进展，就是一个典型的例子。

目标: 新技术对健康专家在治疗 PTSD 的实践上的影响还有待商榷。最新的进展可能干扰了临床实践，以及临床医生本身的信念、道德和表现，乃至对他们的职业文化提出了质疑。在本文中，我们进行了一个宽泛的检索，以便着重标记出那些能更好反映这些新技术的潜力的文章。

方法: 我们在PubMed数据库里使用‘[PTSD] [Posttraumatic stress disorder] AND [Computer] OR [Computerized] OR [Mobile] OR [Automatic] OR [Automated] OR [Machine learning] OR [Sensor] OR [Heart rate variability] OR [HRV] OR [actigraphy] OR [actimetry] OR [digital] OR [motion] OR [temperature] OR [virtual reality] ’作为标题检索词，限制截止时间为2017年7月。

结果: 我们把合成的文献总结成两类：预测和评估（包括诊断、筛选和调控）。两个独立的评审对来源进行筛选、数据抽取和质量评价。对结果进行了综合性叙述。

结论: 这份总结显示出许多进行中的研究，使用生物感受器的被动数据来建立一个 PTSD数码表型。使用生态瞬时评估（EMA）的主动数据可能可以用来评估 PTSD 病人的严重程度。连接对象、人工智能和远程调控的定位仍需定义。这些工具因此需要根据医生和病人的不同情况进行解释和调整。重要的是，让病人、照顾者和其他健康专家参与其中来对这些工具进行设计和评估。

## 1. Introduction

Posttraumatic stress disorder (PTSD) is a severe and frequent disorder. Lifetime prevalence is estimated at 1.3–12.2% (Karam et al., 2014). The probability of developing PTSD after a traumatic event varies according several risk factors (Hoge, Riviere, Wilk, Herrell, & Weathers, 2014, Kessler, Sonnega, Bromet, Hughes, & Nelson, 1995) which can be classified as pre-trauma (sex, IQ, prior trauma exposure, prior mental disorder, genetics, personality factors), related to trauma (perceived fear of death, assaultive trauma, severity of trauma, physical injury) or post-trauma (high heart rate, low social support, financial stress, pain severity, intensive care stay, traumatic brain injury, peritraumatic dissociation, acute stress disorder, disability; Sareen, 2014). Functional and emotional impairments impact on quality of life. There are significant financial and social consequences with elevated rates of hospitalization, suicide attempts and alcohol abuse.

Evolution of PTSD is problematic: remission rates vary at 8–89% (Morina, Wicherts, Lobbrecht, & Priebe, 2014) and 40–50% of patients do not respond or only partially respond to antidepressant treatment (Friedman, Marmar, Baker, Sikes, & Farfel, 2007). Regardless of the chosen treatment technique, about 40% of subjects present a recurrence of symptoms within the year (Martenyi & Soldatenkova, 2006, Tarrier, Sommerfeld, Pilgrim, & Humphreys, 1999) with a risk of relapse estimated at 20% within five years (Boe, Holgersen, & Holen, 2010). Most studies found a weak association of pre-trauma factors with recovery, presumably due to the strong influence of post-trauma factors (Rosellini et al., 2017). This implies that prediction of PTSD evolution based on pre-exposure characteristics would be inefficient: thus, identification of risk factors remains challenging. The prediction and diagnosis of PTSD is a major health issue, particularly regarding the monitoring of soldiers returning from combat or civilians involved in road accidents or physical assaults (including terrorist attacks). It is also a major issue for health professionals: PTSD is often under-recognized due to poor care access and lack of pathology recognition by untrained professionals (Kostaras, Bergiannaki, Psarros, Ploumbidis, & Papageorgiou, 2017; Williams, 2017; Zimmerman & Mattia, 1999).

New technologies can change our way of understanding psychiatric disorders including PTSD. Imaging and biomarkers, along with technological and medical informatics developments, might provide an answer regarding the identification of at-risk patients. Clinicians currently use conventional assessment methods based on systematic collection of information during consultations or from observations reported by others, sometimes using standardized assessment tools (e.g. PCL-5 Posttraumatic Stress Disorder Checklist for DSM-5 ; Ashbaugh et al., 2016). New tools are disrupting this ‘classical’ psychiatry. Recent development of the concept of ‘digital phenotype’ (Torous, Onnela, & Keshavan, 2017) or ‘digital signature of the disease’, which refers to the capture of characteristics of a psychiatric disorder by computerized measurement tools, is one paradigmatic example. Various models of digital phenotype are emerging for schizophrenia (Torous & Roux, 2017) and mood disorders (Bourla, Ferreri, & Ogorzelec et al., 2017). For instance, behaviours or symptoms could be objectified and quantified by computer tools, which would constitute an ‘e-semiotics’: graphorrhoea of patients with manic episodes is replaced by an increase in the number of SMS and psychomotor retardation results in changes in the accelerometer data. These new technologies would therefore make it possible to better assess the mood state of patients with depressive disorders, or to evaluate more finely the disorganization of patients with schizophrenia, while allowing clinicians to gather information remotely and in real time.

In PTSD, detailed evaluation of sleep, avoidance behaviour, intrusive memories or hypervigilance symptoms (including heart rate) are good candidate markers. Miniaturization of sensors and use of smartphones to collect data can be used to refine the diagnosis. Machine learning (ML), a special form of Artificial Intelligence (AI) that classifies data based on a number of variables, allowing the emergence of patterns and groups, can be used to identify at-risk psychiatric patients or patients suffering from disease (Mouchabac & Guinchard, 2013).

The impact of these new technologies on health professionals practice in PTSD cases remains to be determined. These evolutions could disrupt the practices and the practitioners in their beliefs, their ethics and their representations, going as far as questioning their professional culture. In order to inform healthcare practitioners about the possibilities, gaps and future challenges of these new technologies, we conducted an overview of the technologies used for prediction and assessment of PTSD.

## 2. Method

We conducted an overview by querying PubMed database based on the title up to June 2017 with the terms [PTSD] [Post traumatic disorder] AND [Computer] OR [Computerized] OR [Mobile] OR [Automatic] OR [Automated] OR [Machine learning] OR [Sensor] OR [Heart rate variability] OR [HRV] OR [actigraphy] OR [actimetry] OR [digital] OR [motion] OR [temperature] OR [virtual reality]. The following inclusion and exclusion criteria were used to identify studies on PTSD prediction and assessment.

### 2.1. Inclusion criteria

We included studies focusing on PTSD prediction and assessment including the use of:

- e-health applications (i.e. computer-, smartphone-, tablet-based applications)
- wearable device (ECG, smartphone-captor, skin-conductance)
- machine learning.

### 2.2. Exclusion criteria

We excluded those studies:

- not including PTSD symptoms as primary or secondary outcome measure
- dealing with media technology (i.e. television, radio, telephone)
- dealing with treatment or intervention of PTSD.

We focused on the articles that better reflect the potential of these new technologies. Articles related to PTSD treatment using these new tools were not analysed.

## 3. Results

### 3.1. Prediction

Regarding PTSD prediction among military personnel returning from war zones, one current approach is to predict the risk from pre-deployment data, but the results are inconclusive (DiGangi et al., 2013). We found two studies (Karstoft, Galatzer-Levy, Statnikov, Li, & Shalev, 2015, Karstoft, Statnikov, Andersen, Madsen, & Galatzer-Levy, 2015) that applied ML to these population groups with promising results. ML is the subfield of AI that gives ‘computers the ability to learn without being explicitly programmed’ (Arthur Samuel, 1959). It uses two different kinds of classification: ‘supervised’ and ‘unsupervised’. Supervised classification automatically identifies rules from databases constituted of ‘examples’; classically, these are already validated patients with an established diagnosis. With unsupervised classification, in which collected data are not labelled, the objective of the software will be to classify them into homogeneously clustering; this makes it possible to find structures which are not yet known. The coupling of ML with complementary examinations (MRI, EEG) finds patterns in order to classify patients in different groups, which could be useful for screening or for the description of subgroups with a particular phenotype (e.g. patients at risk of relapse).

Karstoft et al. applied a Support Vector Machine (SVM) algorithm to 68 characteristics (demographic characteristics, type of trauma, direct consequences of trauma, multiple scales like PTSD Symptoms Scale, Kessler Psychological Distress Scale, Clinical Global Impressions Scale, etc.) evaluated in 957 trauma survivors (Karstoft et al., 2015). With this method, it was possible to predict the occurrence of PTSD with an AUC (Area Under Curve) of 0.75, which is considered good. The same technique was also used on 561 Danish soldiers deployed in Afghanistan in 2009 (Karstoft et al., 2015). Several variables were studied (demographic characteristics, PTSD-Checklist, Beck Depressive Inventory, Symptom Checklist [SCL], Traumatic Life Events Questionnaire, Intelligence test, Positive and Negative Affect Schedule, etc.) on data obtained before and after deployment (previous trauma and treatment, negative emotions, thought suppression). It was thus possible to predict PTSD with an AUC between 0.84 and 0.88, depending on the data used.

Clark et al. (2014) used a traumatic film shown to healthy volunteers during an fMRI. AI was able to deduce that if the activated regions during the traumatic scenes were associated with the occurrence of intrusions then, for all new subjects, activation of a similar pattern (hyper-responsivity in the amygdala and associated limbic regions) was also predictive of intrusions in almost 70% of cases. Thus, it would be potentially possible to predict the occurrence of intrusions in subjects exposed to a traumatic event by performing functional early imaging coupled with ML. An individualized therapeutic strategy before the onset of the disorder could be proposed.

The relationship between PTSD and heart rate variability (HRV) has been extensively studied (Liddell et al., 2016; Rabellino et al., 2017). HVR is the degree of fluctuation of the interval between two cardiac contractions, dependent on the autonomic nervous system and its sympathetic/parasympathetic balance. This biomarker appears to be a good candidate for PTSD prediction because it is a fairly reliable clue to assess physiological responses related to emotions and stress. In a recent two-phase study (Minassian et al., 2015) involving 2160 soldiers, a pre-deployment HRV assessment was performed via finger photoplethysmography and correlated with a PTSD score. It emerged from this study that prevalence of post-deployment PTSD was higher in participants with high pre-deployment Low Frequencies/High Frequencies (LF/HF) ratios compared with participants who did not have high LF/HF ratios. An additional study on 235 soldiers (Pyne et al., 2016) weighed this result by finding that PTSD Check List (PCL) score prior to deployment strongly influences the prediction. In people involved in road accidents, a decreased HRV during evaluation of patients 48-hours post-accident (obtained with a 24-hour ECG), was an excellent predictor of future PTSD with an AUC up to 0.92 (Shaikh Al Arab et al., 2012).

Finally, Freeman et al. (2014) investigated whether the way to respond to a virtual reality (VR) environment predicted the severity of PTSD. To do this, 106 patients who underwent physical assault in the previous month were submitted to an immersive VR experiment and then completed questionnaires (Positive and Negative Symptoms Scale [PANSS], PTSD Symptom Scale–Interview [PSSI], Green et al. Paranoid Thought Scales [GPTS], Posttraumatic Diagnostic Scale [PDS], etc.). This study showed that responses to VR predicted the severity of paranoia and PTSD symptoms as assessed by standard measures six months later.

### 3.2. Assessment

#### 3.2.1. Diagnosis of PTSD

Several technologies can be used to refine the diagnosis of PTSD in patients using imaging data, computer or smartphone questionnaires, or biometric data (sleep, HRV, skin conductance) using connected objects. Coupling MRI and ML makes it possible to distinguish a patient with PTSD from a patient without PTSD with 92.5% accuracy (Liu et al., 2015). Moreover, it is clear that smartphone or tablet evaluation is as accurate as an evaluation by a trained clinician (Price, Kuhn, Hoffman, Ruzek, & Acierno, 2015), with the possibility of making this evaluation much quicker (Finkelman et al., 2017). It is also possible to evaluate PTSD severity according to the patient’s performance in a virtual world. The patient directs an avatar confronted with elements potentially triggering his symptoms (Myers et al., 2016) with the possibility of live monitoring physiological function (Costanzo et al., 2014; Webb, Vincent, Jin, & Pollack, 2015; Wiederhold, Jang, Kim, & Wiederhold, 2002). Indeed, skin conductance, already correlated in many studies (Blechert, Michael, Grossman, Lajtman, & Wilhelm, 2007, Bryant, Harvey, Gordon, & Barry, 1995) with PTSD, may be used as a diagnostic tool (Hinrichs et al., 2017) as it appears correlated with PTSD intensity. However, the capability of VR to reproduce traumatic events or to trigger symptoms with sufficient power is questioned (Van’T Wout, Spofford, Unger, Sevin, & Shea, 2017).

Regarding biometric data, it appears that changes in HRV were significantly associated with PTSD (Hauschildt, Peters, Moritz, & Jelinek, 2011; Woodward et al., 2009), especially during sleep. Several studies (Green et al., 2016; Moon, Lee, Kim, & Hwang, 2013; Norte et al., 2013; Rissling et al., 2016; Wahbeh & Oken, 2013) found a decrease in HRV in patients with PTSD, pointing out that PTSD symptoms may be related to decreased parasympathetic control, especially during sleep (which could constitute a state of vulnerability for decreased parasympathetic cardiac control) (Kobayashi, Lavela, & Mellman, 2014). Other studies demonstrated that low HRV as a sign of over-reactivity to stress were present prior to the development of PTSD (Eraly et al., 2014; Minassian et al., 2015).

Combined data could lead to the development of the digital phenotype of PTSD, a moment-by-moment quantification of the individual-level human phenotype using passive data (GPS, accelerometer, voice, call logs, text logs, screen use) from digital devices (smartphone, wearable devices).

#### 3.2.2. Screening of PTSD

Early detection (i.e. screening) of PTSD is a major public health issue. He et al. (He, Veldkamp, Glas, & de Vries, 2017) applied ML techniques to text mining to detect PTSD with excellent accuracy (82%). Text mining refers to a set of computational processes consisting in extracting knowledge according to certain criteria defined in texts, which makes it possible to model data from linguistic theories. This technique is commonly used for filtering communications (anti-spam filter) and by search engines.

A study (Wolff, Gregory Chugo, Shi, Huening, & Frueh, 2015) comparing computer-administered interviewing (CAI) versus orally-administered interviewing (OAI) showed an excellent correlation between the two evaluation methods. Russo, Katon, and Zatzick (2013) proposed screening Electronic Medical Record (EMR) data to identify predictive factors for the development of PTSD in 878 randomly selected injured trauma survivors. Risk factors were identified using logistic regression, sensitivity, specificity, predictive values and receiver operating characteristic (ROC) curve analyses. Studies show that automated EMR screening can be used to efficiently and accurately sort injury survivors at risk for the development of PTSD.

Several studies proposed centralizing these evaluations in a cloud powered by clinical data, Ecological Momentary Assessment (EMA) on smartphones and data obtained through a website or voice-based automated Tele-PTSD Monitor (Xu et al., 2012). The acquired voice data is sent to a secure server to implement the PTSD Scoring Engine (PTSD-SE) where a PTSD mental health score is computed. If the score exceeds a predefined threshold, the system will notify clinicians (via email or short message service) for confirmation and/or an appropriate follow-up assessment and intervention. This offers an average detection accuracy of 95.9%.

#### 3.2.3. Monitoring of PTSD

PTSD diagnosis features have been tested with monitoring, but most promising is evaluation by EMA. The evaluation of symptoms daily, in the habitual environment of the patient, free from recall biases, as the patient self-assesses '*right then, not later; right there, not elsewhere*' (Csikszentmihalyi & Larson, 1987).

Possemato et al. (2015) assessed the relationship between alcohol use and the intensity of PTSD by EMA. The authors reported an accentuation of alcohol use as a function of PTSD severity, particularly overnight. This method can also be used to evaluate intrusions in real time (Kleim, Graham, Bryant, & Ehlers, 2013). Patients are relatively compliant with the use of this type of method (63.1–86% response rate) (Possemato et al., 2012; Price et al., 2014).

## 4. Discussion

This overview highlights the diversity of new technologies used in psychiatry and their application to the prediction, diagnosis and follow-up of PTSD. In terms of diagnosis, biometric data associated with PTSD are beginning to emerge. The digital phenotype (Torous et al., 2017) of PTSD is not yet clearly determined, despite good agreement of the following data:

- decreased HRV
- dissociation between ANS activity and total sleep time
- increased skin conductance correlated to PTSD intensity.

We noted the absence of studies using GPS or call log data (Faurholt-Jepsen et al., 2014; Saeb et al., 2015), thus underlining the specificity of digital markers according to the pathologies studied: sleep and signs of neurovegetative hyperactivation (HRV modifications) in PTSD, study of psychomotor behaviour and verbal flow in thymic disorders. However, GPS data could be relevant to evaluate behaviour of avoidances. Data analysis from phone conversation or SMS could allow aquantification of traumatic related-terms.

PTSD screening in patients exposed to trauma benefit from the contribution of new technologies, and two very different methods emerge. First, a direct approach is to increase the interviewing capacity of at-risk patients using computerized questionnaires. This method is reminiscent of the recent development of Computerized Adaptive Testing (CAT) (Gibbons et al., 2008): a computer-administered test in which the next item or set of items selected to be administered depends on the correctness of the test taker’s responses to the most recent items administered. It aims to mimic the functioning of the clinician using a limited AI that will adapt the questions automatically to patient answers using a database containing a large number of possible questions. These computerized questionnaires, for example, can be used to diagnose depression (Gibbons et al., 2012). Some related systems use ‘avatars’ of psychiatrists able to converse directly with patients: the Embodied Conversational Agent (ECA) (Philip et al., 2017). Second, an indirect approach consists of the analysis of data (EMR, text) using AI (text-mining, SVM, Product Score Model [PSM]) or statistics (logistic regression).

In terms of monitoring, EMA techniques appear promising for evaluating the evolution of day-to-day symptoms. Biometric markers and computer translation give the possibility of doing it remotely, and in a way that is not intrusive, with sometimes better accuracy than a clinician. Collecting data in psychiatry involves a classic interview: the psychiatrist observes, interrogates and evaluates the patient in order to form an opinion on a probable diagnosis. It is only recently that this clinical examination can be enhanced by ‘active’ data collection. The patient is involved in the evaluation of his own symptoms at regular intervals and in his usual environment. Studies showed that EMA applications are as reliable as the scales usually used for PTSD evaluation (PCL in particular), which has already been demonstrated for the assessment of depression (QIDS, PHQ9, BDI) with excellent acceptability, or even preference for this medium, by the patients, in particular with regard to the suicidal dimension (Torous et al., 2015). It seems that some patients are more willing to confide their dark feelings to their phone than to their doctor.

Moreover, this repeated evaluation of symptoms over time may in itself have a therapeutic effect. A study conducted with bipolar patients (Faurholt-Jepsen et al., 2015) found that this could potentially limit manic or hypomanic episodes. The feeling of intrusion that can be caused by the self-questionnaires is relatively little reported by these studies, which highlights the good acceptability and the good compliance of the patients.

In terms of prediction, coupling imaging examinations (functional MRI) with ML will probably in the short term predict the risk of developing PTSD after a traumatic exposure. These techniques can correctly classify a patient according to his mood state with an accuracy up to 92% (Wu et al., 2017). In the field of psychotic transition, these techniques predict with 84.2% accuracy the risk of transition from an at-risk mental state to schizophrenia (whereas a trained clinician have less than 50% accuracy) (Koutsouleris et al., 2012).

This overview is the first to attempt to outline the future of PTSD evaluation using new technologies. A review suggesting guidelines on new technologies in the treatment of PTSD has already been proposed by Gaebel et al. (2017). Our article makes it possible to highlight a number of limitations:

- studies focus on specific populations: veterans, men, youth, etc.
- small sample size
- impact of multiple measurements via a mobile device and the resulting fatigue
- recruitment bias
- some statistical weaknesses
- absence of gold standard in a number of cases (polysomnography for the study of sleep, no clinician administered clinical PTSD diagnosis).

These limitations reveal that this is a new field of experimentation which requires guidelines to allow the implementation of studies with better evidence level.

All these evolutions disrupts the ways of thinking and practicing psychiatry, and thus question the psychiatrists’ ‘professional culture’. This sociological notion raises the fact that professionals refer not only to a linked set of theoretical knowledges and technical ‘recipes’ learned or accumulated with experience, but that they share a specific language and common value. Psychiatrists diverge from other medical specialties due to the predominance of clinical reasoning, the lack of specific or valid imaging techniques or biological tests, and the importance given to intuition, clinical sensitivity and therapeutic relationship. The psychiatry clinic appears to be challenged by the emergence of techniques that profoundly modify clinical data collection. In our recent study questioning 512 psychiatrists about these new technologies, about 25% of psychiatrists are frankly refractory, 50% are expecting, while 25% are enthusiastic about increasing their capacity with these new systems (Bourla, 2017). This medical, ethical and legal debate thus enriches an in-depth reflection on the professional culture of physicians in general and of psychiatrists in particular.

Similarly, the question of patients’ acceptability, implementation in health care and the cost effectiveness of these new technologies remain under debate. There is an urgent need for well-designed clinical and medico-economic studies

## 5. Conclusion

This overview shows that many studies are underway and that it is possible to start building the PTSD digital phenotype using passive data obtained by biometric sensors (skin conductance and HRV in particular). EMA would allow clinicians to assess active data obtained from PTSD patients (intrusion, sleep, etc.) to get an idea of their severity. The place of connected objects, AI and remote monitoring of patients with psychiatric pathology remains to be defined, and the question of data security will quickly become central. In order to prevent data from being used for other than medical purposes, we believe that it is essential that physicians take up this issue and make recommendations on the subject. Important ethical and deontological considerations hamper the acceptance of these technologies, which seem to be strongly conditioned by the degree of ‘scientificity’ of psychiatrists: the more they are, the more they accept these new technologies (Bourla, 2017). To be used, these tools must therefore be explained and adapted to the different profiles of physicians and patients while taking into account the risks inherent in their use (data piracy, false positives, risk, etc.). The involvement of patients, caregivers and other health professionals is essential to design and evaluate these new tools.
